# Bridging electrocatalyst and cocatalyst studies for solar hydrogen production *via* water splitting

**DOI:** 10.1039/d1sc06015e

**Published:** 2022-02-08

**Authors:** Masaki Saruyama, Christian Mark Pelicano, Toshiharu Teranishi

**Affiliations:** Institute for Chemical Research, Kyoto University Gokasho, Uji Kyoto 611-0011 Japan saruyama@scl.kyoto-u.ac.jp teranisi@scl.kyoto-u.ac.jp

## Abstract

Solar-driven water-splitting has been considered as a promising technology for large-scale generation of sustainable energy for succeeding generations. Recent intensive efforts have led to the discovery of advanced multi-element-compound water-splitting electrocatalysts with very small overpotentials in anticipation of their application to solar cell-assisted water electrolysis. Although photocatalytic and photoelectrochemical water-splitting systems are more attractive approaches for scaling up without much technical complexity and high investment costs, improving their efficiencies remains a huge challenge. Hybridizing photocatalysts or photoelectrodes with cocatalysts has been an effective scheme to enhance their overall solar energy conversion efficiencies. However, direct integration of highly-active electrocatalysts as cocatalysts introduces critical factors that require careful consideration. These additional requirements limit the design principle for cocatalysts compared with electrocatalysts, decelerating development of cocatalyst materials. This perspective first summarizes the recent advances in electrocatalyst materials and the effective strategies to assemble cocatalyst/photoactive semiconductor composites, and further discusses the core principles and tools that hold the key in designing advanced cocatalysts and generating a deeper understanding on how to further push the limits of water-splitting efficiency.

## Abstract 2

This perspective briefly reviews recently developed water splitting electrocatalyst materials and discusses their utilization as cocatalysts for photocatalytic and photoelectrochemical water splitting systems.

## Graphical abstract

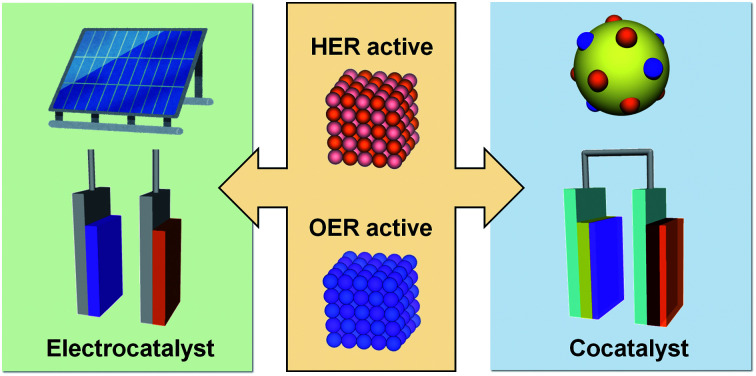

## 1. Introduction

Reducing the amount of CO_2_ emitted from burning fossil fuels is essential to mitigate global warming.^1^ To meet this challenge while addressing the growing global energy demand, it is becoming increasingly important to develop sustainable, carbon-neutral energy sources.^2^ Molecular hydrogen (H_2_) is regarded as an ideal green fuel because it releases zero emissions and only produces water upon combustion.^3^ Therefore, H_2_ is expected to hold prime significance as a high-energy-density fuel in future energy systems. In fact, H_2_ fuel cells have been implemented to power vehicles with advanced H_2_ transportation technologies.^4^

Although hydrogen is the most abundant element on earth, the dihydrogen molecule rarely exists in nature; therefore, H_2_ must be produced artificially. Currently, steam reforming is the preferable method for producing commercial H_2_; however, a substantial amount of energy is required to drive this process and CO_2_ is emitted as a byproduct.^5^ Recently, sunlight-driven water splitting has emerged as an attractive approach for green and sustainable H_2_ production.^6^ Among solar-hydrogen technologies, water electrolysis using electricity generated from solar cells is considered to be the most advanced pathway.^7^ Combining a solar cell with an electrocatalyst (EC)-loaded electrode can promote water splitting, reaching >30% solar-to-hydrogen efficiency (STH) using an InGaP/GaAs/GaInNAsSb triple-junction solar cell.^8^

Thermodynamically, the reduction and oxidation of water start at 0 V_RHE_ and +1.23 V_RHE_, respectively, meaning that the overall water-splitting reaction can start from 1.23 V. However, the activation energy for each half-reaction contributes to an overpotential, which requires an additional voltage (hundreds of millivolts) to overcome and drive the water-splitting process.^9^ In practical applications, ECs are employed to reduce this overpotential, thereby improving the STH.

To date, the high cost of device manufacturing has hindered the practical implementation of solar cell-assisted H_2_-production devices. However, the direct decomposition of water through photocatalysis represents a promising alternative approach owing to its technical simplicity and low associated investment costs.^10,11^ Scaling up a photocatalytic system is also relatively much easier because water splitting can proceed by simply immersing semiconductor photocatalyst (PC) powder in water under light irradiation.^12^ Recently, a large-scale experiment (100 m^2^ scale) demonstrated that immobilizing powdered PCs on panels could lead to the production of about 600 L of H_2_ on a sunny day.^13^ Such photoactive semiconductors can also be used as light-absorbing layer of photoelectrodes (PEs) for photoelectrochemical (PEC) water-splitting cells.^14^ These electrodes are usually placed in separate compartments which are electrically connected through an external circuit. Although this configuration makes PEC systems more complex in terms of design, higher STH efficiencies have been achieved due to easier product collection and elimination of potential back-reactions.^15^ For example, a tandem-type PEC cell reached an STH of >7%, which far exceeds current STH values for PC systems.^16^ However, both PC and PEC systems still have large rooms to reach a target STH of 10% with a long-term durability for practical application.^12^

To realize a higher water-splitting performance, substantial efforts have been mainly devoted to the development of semiconductors as efficient photoactive materials.^10^ Cocatalysts (CCs), which are water-splitting catalysts coupled with PCs or PEs, also have been systematically studied as equally-important components in both PC and PEC systems.^17^ Because the semiconductor surface tends to have a weak driving force for redox reactions involving water, coupling with CCs can facilitate these redox reactions by lowering the activation energy barrier.^17^ Specifically, charged-up CCs retain suitable steady-state potentials to drive the surface reactions by serving as trapping sites for photogenerated carriers (*i.e.*, electrons and holes). In turn, this process not only promotes charge separation but also suppresses adverse charge recombination and photocorrosion.^18,19^

While the integration of CCs with PCs and PEs is indispensable for achieving excellent efficiencies, the CC materials have been explored much less than the EC materials. The straightforward application of previously-reported ECs materials as CCs is challenging because it introduces various factors that must each be critically considered.^18^ First, CCs should be small or thin enough so that they do not limit the amount of light absorbed by the PC and PE.^20^ Second, the change in potential shift at the CC/semiconductor junction is critical for evaluating charge transfer efficiency.^21^ These additional requirements have not yet been sufficiently addressed, delaying advancement related to CC materials.

**Fig. 1:**
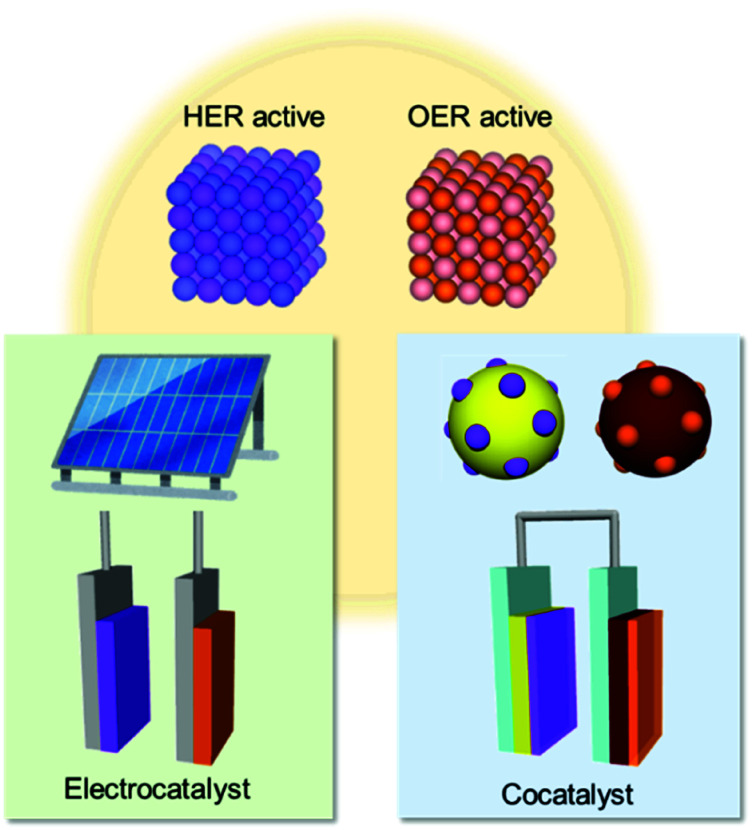
Application of materials active for solar-driven water splitting *via* the hydrogen evolution reaction (HER) and oxygen evolution reaction (OER).

In this perspective, we discuss the recent advances in the design of EC materials and the strategies for constructing CC/PC and CC/PE composites, including our group's nanoparticle (NP)-adsorption approach. A special emphasis is put on the key principles governing the rational assembly of an efficient CC/PC and CC/PE system. We hope this perspective will inspire researchers in pursuit of bridging the gap between the developments of highly-active EC and CC materials, which have only been studied separately to date (Fig. 1).

## 2. Electrocatalysts for water electrolysis

### 2.1 Development of electrocatalyst materials

Water splitting involves two concurrent catalytic half-reactions: the hydrogen evolution reaction (HER) and the oxygen evolution reaction (OER). Materials that can lower the activation energies of these reactions are utilized as ECs. Although noble metals such as Pt, Ru(O_*x*_) and Ir(O_*x*_) are still representative efficient ECs for water splitting, diverse earth-abundant compounds with comparable catalytic activities have also been developed based on comprehensive theoretical and experimental studies.^22^ Herein, we briefly introduce recently-developed EC materials for efficient water splitting and discuss their typical preparation methods. A comprehensive introduction of EC materials is beyond the scope of this perspective; thus, we encourage the interested reader to refer to recent reviews summarizing the development of EC materials.^23,24^

#### 2.1.1 HER electrocatalysts

**Fig. 2:**
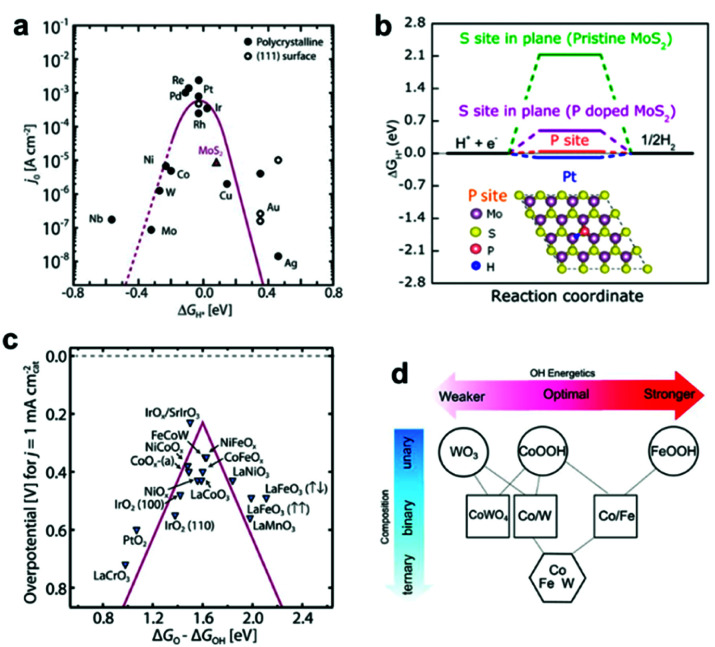
(a) Volcano plot relating the ECs' HER activities and Δ*G*_H*_; (b) HER free–energy diagram for P and S sites in the basal plane of pristine and P-doped MoS_2_; (c) OER volcano plot for metal oxides; (d) OH adsorption energetics (Δ*G*_OH*_) as a function of the material's composition obtained by interpolation between the calculated pure phases of WO_3_ (001), CoOOH (01−12), FeOOH (010), and CoWO_4_ (010) planes. Adapted with permission from (a and c) ref. 22, (b) ref. 27, and (d) ref. 44; copyright 2017 American Association for the Advancement of Science (AAAS), 2017 the American Chemical Society (ACS), and 2016 AAAS, respectively.

Platinum (Pt) is a traditional and benchmark HER EC. Theoretically, the binding energy of an H atom on the EC surface in an intermediate state (defined as Δ*G*_H*_) is widely accepted as an index in the selection of an EC for HER.^22^ It is believed that ECs with Δ*G*_H*_ = 0 are most suitable for efficient HER, and Pt has the Δ*G*_H*_ closest to 0 among all elements.^25^ From this perspective, materials based on the combination of other elements can be a promising replacement for Pt (a precious metal) if their Δ*G*_H*_ is close to 0.^22^ Significant research efforts have uncovered new combinations of earth-abundant elements that exhibit good HER activities (Fig. 2a).^23^ MoS_2_ was a pioneering EC material^26^ that launched successive explorations into HER-active 2D metal dichalcogenides. With support from theoretical studies, considerable enhancements of the HER activities of MoS_2_ compounds have been demonstrated by introducing defects and elemental dopants (Fig. 2b).^27,28^ Metal (Ni, Co, Fe, W, Mo, …) phosphides, nitrides, and carbides are also subjects of extensive studies owing to their advantageous electronic structures.^27,29–38^ Tuning the metal composition has proven to be pivotal in realizing exceptional HER performance.^29^ Such rationally designed EC compounds have realized comparable HER activities over a wide range of pH for practical applications.

#### 2.1.2 OER electrocatalysts

Although noble metal-based IrO_*x*_ and RuO_*x*_ materials remain as the state-of-the-art ECs for OER,^39,40^ earth-abundant 3d transition metal-based materials have attracted attention owing to their excellent OER activities (*i.e.*, NiFeOOH).^41,42^ Computational and experimental evidence have shown that the difference between the adsorption energies of the O* and OH* intermediates (*i.e.*, Δ*G*_O*_ − Δ*G*_OH*_) is a reliable indicator for predicting the OER activity of an EC (Fig. 2c).^43^ For example, Sargent *et al.* used theory and experiments to establish that an exceptional OER activity can be attained by tailoring the material's composition in terms of three metals (Co, Fe, and W), which enables an optimization of Δ*G*_OH*_ (Fig. 2d).^44^ In addition, researchers have discovered that chalcogenides and phosphides containing two or more types of metals can also function as excellent OER ECs.^44–54^

#### 2.1.3 Bifunctional compounds as electrocatalysts

While exploring new EC materials, researchers have identified various compounds that are highly-active for both HER and OER. For example, multi-metallic compounds, such as oxides, phosphides, and chalcogenides, achieve efficient overall water splitting in a two-electrode configuration driven by a cell voltage of ∼1.6 V in alkaline electrolyte (Table 1).^48,55–60^ Higher pH conditions (alkaline media) are usually more appropriate for such bifunctional materials because they facilitate the rate-determining OER, which has a larger overpotential than the HER.^61^ Bifunctional EC materials represent promising CCs for photocatalytic water splitting owing to their inherent abilities to promote HER and OER simultaneously.

**Table 1:** Selected recently-developed multi-metallic EC materials and their performance compared with conventional EC^a^

EC	Preparation method	Catalysis	Overpotential (mV for 10 mA cm^−2^)	Electrolyte	Ref.
P-doped MoS_2_	Hydrothermal	HER	43	0.5 M H_2_SO_4_	27
Re_1–*x*_Mo_*x*_Se_2_	Hydrothermal	HER	77	0.5 M H_2_SO_4_	34
Fe_0.5_Co_0.5_P	Hydrothermal + phosphidation	HER	37	0.5 M H_2_SO_4_	33
20w% Pt/C	Commercial	HER	27	0.5 M H_2_SO_4_	34
Fe_3_GeTe_2_	Solid-state reaction	HER	105	1 M KOH	35
Ni_2_Mo_3_N	Nitridation	HER	21.3	1 M KOH	36
Ni_6_Mo_6_C/NiMoO_*x*_	Hydrothermal + carburization	HER	29	1 M KOH	37
20w% Pt/C	Commercial	HER	32	1 M KOH	37
G-FeCoW	Sol–gel	OER	191	1 M KOH	44
NiCoFe–P–NP@NiCoFe–PBA	Precipitation + phosphidation	OER	223	1 M KOH	49
(CrMnFeCoNi)S_*x*_	Pulse thermal decomposition	OER	116	1 M KOH	51
CoVFeN	Hydrothermal + electrodeposition + nitridation	OER	212	1 M KOH	53
Mo_6_Ni_6_C	Hydrothermal + carburization	OER	190	1 M KOH	54
RuO_2_	Commercial	OER	264	1 M KOH	49
IrO_2_	Commercial	OER	290	1 M KOH	54
NiFe LDH	Electrodeposition	OER	250–300	1 M KOH	52
RuIr	Chemical reduction	OWS	255	0.05 M H_2_SO_4_	56
SrNb_0.1_Co_0.7_Fe_0.2_O_3−*δ*_	Solid-state reaction	OWS	450	1 M KOH	55
MoS_2_/Ni_3_S_2_	Solvothermal	OWS	330	1 M KOH	57
NiMoP NSs@MCNTs	Hydrothermal	OWS	369	1 M KOH	58
Ni–Fe–MoN	Solid-state reaction	OWS	228	1 M KOH	59

a OWS = overall water splitting.

### 2.2 Synthesis and use of electrocatalysts

**Fig. 3:**
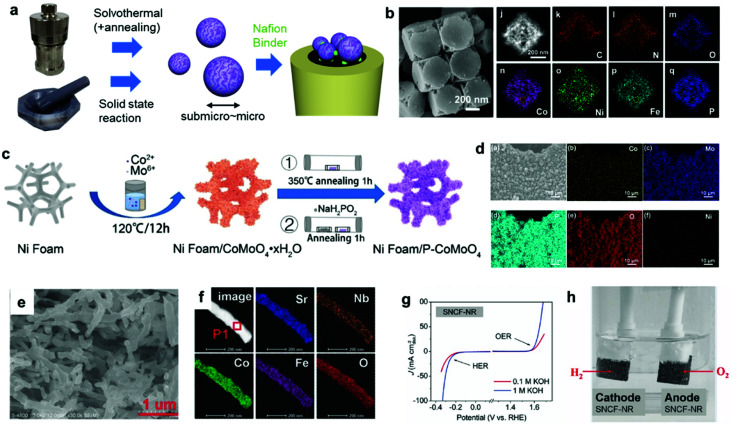
(a) Typical synthetic strategies for powdered ECs and the corresponding electrode preparation; (b) MOF-derived NiCoFe–P NPs containing porous nanocages; (c) direct EC (P–CoMoO_4_) growth on a Ni foam electrode through a solvothermal method and phosphidation *via* annealing; (d) elemental mapping of a Ni foam/P–CoMoO_4_ composite electrode; (e) scanning electron microscopy (SEM) and (f) elemental mapping images, (g) CV, and (h) overall water-splitting experiment using a bifunctional SrNb_0.1_Co_0.7_Fe_0.2_O_3−*δ*_ EC. Adapted with permission from (b) ref. 49, (c and d) ref. 38, and (e–h) ref. 55; copyright 2021 ACS, 2020 Wiley, and 2017 Wiley, respectively.

To date, innovative EC materials have been synthesized through various chemical and physical routes. Because the overall size of the ECs does not require fine-tuning (as long as they are conductive enough to work on electrodes), they have mainly been fabricated using facile strategies, including solvothermal syntheses, solid-state reactions, and electrochemical deposition (Fig. 3a). This enables rapid and widespread exploration of novel EC materials *via* screening experiments.^62^ Simple oxides and chalcogenides can be directly synthesized by liquid-phase methods under mild conditions,^62,63^ whereas high-temperature annealing is often required to obtain phosphides, nitrides, and carbides from their corresponding preformed oxides.^34–36,51,52^ Specifically, they are annealed with precursors (P = NaH_2_PO_2_; N = NH_3_; C = organic molecule) in a furnace at >300 °C for complete conversion. To assess the resulting material's electrocatalytic activity, powdered ECs are embedded on a conductive carbon substrate, and the composite is typically glued to an electrode surface using a Nafion binder.^64^ ECs can also be grown directly on porous conductive supports, such as metal foam or carbon paper/cloth, which increase their conductivity and loading per geometrical unit area (Fig. 3c and d).^37,38,65^ Such EC-grown substrates can be directly connected to a potentiostat as free-standing electrodes. Alternatively, nanosizing is a practical approach for designing active ECs with large surface areas because it provides unsaturated surface atoms and promotes greater atomic usage efficiency.^23^ Effective techniques for fabricating EC nanoparticles (NPs) include colloidal synthesis and precursor decomposition on a support material.^51,66^ Annealing a metal–organic framework (MOF) is a facile technique to produce multi-metallic NPs supported on a porous conductive carbonaceous substrate (Fig. 3b).^49^ Moreover, recent advances have revealed that single-atom (SA) catalysts are emerging as new ECs with exceptional properties, such as maximum atomic usage efficiency, high activity of the metal complex, and high durability of the bulk catalyst.^67^ Similar to powdered ECs, EC NPs and SA catalysts can be supported on conductive substrates and fixed to an electrode. These EC electrodes operate as working electrodes in a three (working, reference, and counter)-electrode system to evaluate their catalytic performance towards HER and/or OER.^68^ Overall water splitting is investigated in a two (working + counter)-electrode configuration using two identical EC-immobilized electrodes (Fig. 3h).^55^

**Fig. 4:**
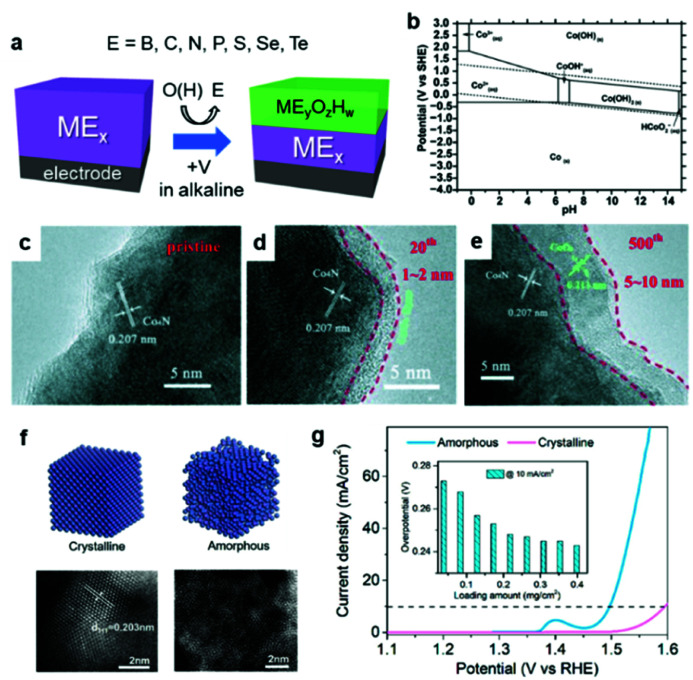
(a) Schematic diagram illustrating the *in situ* oxidation of an EC; (b) Pourbaix diagram of Co–H_2_O system; (c–e) transmission electron microscope (TEM) images of the Co_4_N EC surface before and after 20 and 500 cycles of CV for OER; (f) structural differences between crystalline and amorphous NiFe and (g) their corresponding OER polarization curves. Adapted with permission from (b) ref. 70, (c–e) ref. 71, and (f and g) ref. 72; copyright 2019 the Royal Society of Chemistry (RSC), 2015 Wiley, and 2020 ACS, respectively.

It is worth noting that ECs can undergo some changes during catalysis, especially in a OER environment.^69^ According to the Pourbaix diagram, the surface or entire EC can transform into its corresponding [(oxy)hydr]oxide under alkaline conditions under a positive applied potential (Fig. 4a–e).^70^ Wu *et al.* reported that the surface of Co_4_N was progressively oxidized during OER cyclic voltammetry (CV) experiments in 1 M KOH electrolyte.^71^ Even if the composition is carefully controlled during synthesis, the real active species may deviate from the as-prepared material. Such transformed oxides and (oxy)hydroxides frequently exhibit superior catalytic activities because their amorphous surfaces offer more active sites and enhanced electronic interactions relative to their pure oxide analogs (Fig. 4f and g).^72^ In calculations of the surface state, a lack of understanding of the real active species may mislead research conclusions.

## 3. Cocatalysts for PC and PEC systems

In the case of solar cell-assisted water splitting, EC-loaded electrodes can be employed in the same configuration as two electrode system. Therefore, improving intrinsic catalytic activity of EC directly increases the STH, aside from the solar cell efficiency. In terms of PC and PEC systems, the latest advancements in semiconductor design (*e.g.*, defect engineering and morphology control) have paved the way for advanced PCs and PEs with high light-energy conversion efficiencies.^73^ Nevertheless, additional considerations concerning the successful integration of efficient EC materials as CCs onto the PC and PEC systems must be addressed. First, it is essential for the CCs to have suitable NP dimensions and homogenous distribution on the PC and PE to ensure that they do not block the incident light. Second, designing an optimal interface between the semiconductor and CC is necessary because the stability and performance of the entire system largely depend on this junction. Such requirements significantly limit the range of material dimensions (*e.g.* size, shape, and light absorbance.) that can be viewed as valuable for CCs, thereby hindering further development of CC research compared with EC research.

**Fig. 5:**
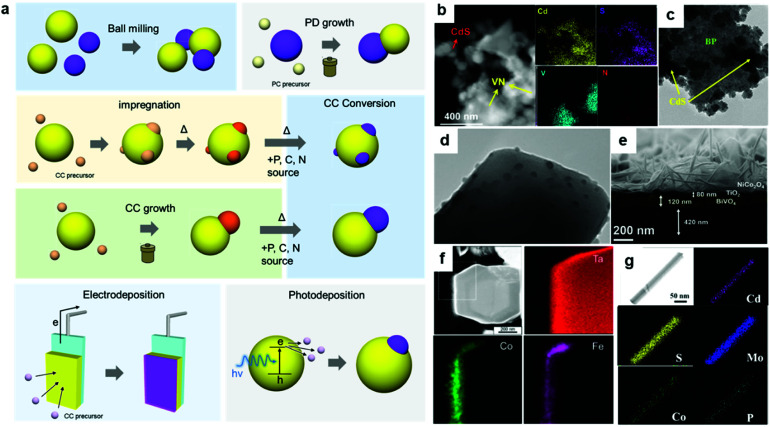
(a) Representative strategies for assembling CC/PC composites; (b) VN loaded on CdS *via* ball-milling; (c) CdS grown on BP *via* a hydrothermal method; (d) Ca_2_FeCoO_5_ loaded on TiO_2_*via* impregnation; (e) NiCo_2_O_4_ grown on a TiO_2_/BiVO_4_ photoelectrode *via* a hydrothermal method; (f) CoO_*x*_-FeO_*x*_ coated on Ta_3_N_5_:Mg + Zr *via* electrodeposition; (g) MoS_2_ and CoPi loaded on CdS nanowires *via* photodeposition. Adapted with permission from (b) ref. 80, (c) ref. 87, (d) ref. 101, (e) ref. 127, (f) ref. 134, and (e) ref. 122; copyright 2019 ACS, 2020 ACS, 2020 Wiley, 2020 ACS, 2015 ACS, and 2019 ACS, respectively.

Despite these difficulties, a significant amount of effort has been dedicated to the hybridization of water-splitting catalyst materials with semiconductors to realize high-performance photocatalytic and PEC systems.^74^ Unlike in the synthesis of ECs, the stability of PCs and PEs must be considered when constructing CC/semiconductor composites. PCs based on (oxy)nitride and (oxy)chalcogenide decompose easier than pure oxides, while other PCs become inactive because of a change in the metal valence.^75^ As introduced in Section 2, some methods for efficient EC formation require high-temperature annealing, and such a harsh condition cannot be applied to unstable semiconductors. In this section, we summarize representative strategies for loading CCs onto PCs and PEs (Fig. 5a, Tables 2 and 3).

**Table 2:** AQY of powdery CdS PC/CC prepared by various hybridization methods

CC	Loading method	AQY (% @ 420 nm)	Sacrificial reagent	Ref.
VN	Sonication	5.3	Lactic acid	80
P-doped Ni_2_S	Sonication	4.8	Na_2_S + Na_2_SO_3_	81
WP	Ball-milling	10.5	(NH_4_)_2_SO_3_	82
Co_3_C	Mixing + annealing	17	Na_2_SO_3_ + Na_2_S	86
FeSe	Sonication + annealing	6.71	Na_2_S + Na_2_SO_3_	85
MoS_2_/C	PC growth	28.1	Lactic acid	91
Co_3_S_4_/Co@C	PC growth	∼15	Na_2_S + Na_2_SO_3_	92
Ni_3_S_2_	PC growth	7.86	Lactic acid	93
Mo_2_N	PC growth	2.2	Na_2_S + Na_2_SO_3_	94
WS_2_	Impregnation	5.0	Lactic acid	98
CoO	Impregnation	40.64	Lactic acid	99
NiS	Hydrothermal	74.1	Lactic acid	106
MoS_2_	Hydrothermal	41.37	Lactic acid	107
NiCoP/NiCoPi	Hydrothermal + phosphidation	45	Lactic acid	111
β-Ni(OH)_2_	Photodeposition	72	Ethanol	116
WS_*x*_	Photodeposition	14.7	Lactic acid	120
CoPi	Photodeposition	24.3	Lactic acid	121
MoS_2_-CoPi	Photodeposition	36	Lactic acid	122

**Table 3:** Examples of anodic PEs loaded with CC in various methods

Semiconductor	CC	CC loading method	Photocurrent density (mA cm^−2^ @ 1.23 V)	Ref.
BiVO_4_	FeSnOS	Impregnation	3.1	124
BiVO_4_/TiO_2_	NiCo_2_O_4_	Hydrothermal	2.47	127
BiVO_4_	NiFeY LDH	Hydrothermal	5.2	128
BiVO_4_	F:Co(CO_3_)_*x*_OH_*y*_	Hydrothermal	5.5	129
BiVO_4_	Co–LaFeO_3_	Electrodeposition	3.4	135
BiVO_4_	FeOOH/NiOOH	Light-assisted electrodeposition	4.2	136
BiVO_4_	NiP_*x*_@FeP_*y*_O_*z*_	NP adsorption	2.3	148
BiVO_4_	CuCoO_2_	NP adsorption + annealing	3.32	126
Ta_3_N_5_	FeNiO_*x*_	Impregnation	9.95	125
Ta_3_N_5_	FeNiCo	Electrodeposition	4.0	133
Ta_3_N_5_	Co–Pi/Co(OH)_*x*_/NiFe-LDH	Adsorption + electrodeposition + solvothermal	6.3	137
Ta_3_N_5_	Ni:CoFeO_*x*_	Light-assisted electrodeposition	5.3	138

### 3.1 Cocatalysts loading on powdery photocatalysts

In photocatalysis using powdery PCs, both reduction and oxidation reactions take place on an individual PC particle, requiring two suitable surfaces on an PC particle to drive each particular half-reaction. Generally, CCs for a specific half-reaction are first loaded onto the PC surface leaving the other regions available to facilitate the other half-reaction, which is especially advantageous for examining individual half-reactions including the cases using sacrificial reagents. For overall water-splitting PC, the loading of CCs for both HER and OER can further accelerate the total reaction to attain higher photocatalytic performance.^76^

#### 3.1.1 Hybridizing photocatalysts with cocatalysts

Mechanical mixing is the simplest way to modify a PC with an CC. Ultrasonication and (ball-)milling (a physical mixing method) are useful for preparing a well-mixed powdery composite.^77–82^ Thermal treatment application is often necessary to strengthen the interfacial contact after mixing.^83–86^ The main advantage of this technique is that it does not impose any restrictions on the type of PC or CC that can be used; thus, a wide range of metal alloys, phosphides, nitrides, borides, carbides, and chalcogenides can be employed. For example, simple ball-milling can yield a CdS/VN composite without decomposing either constituent (Fig. 5b).^80^

Chemical methods are also applicable to hybridize PC with synthetic CC materials. Adding a CC to a PC synthetic system can grow typical PCs, such as CdS, on the CC surface under mild hydrothermal conditions (Fig. 5c).^87–94^ However, this technique is not viable for bulk oxide-based PCs (*e.g.*, SrTiO_3_) which require high-temperature (>800 °C) solid-state synthetic reactions that can lead to CC decomposition.

#### 3.1.2 Cocatalyst growth and transformation on photocatalysts

A simple route to deposit a nanoparticulate metal oxide CC on a PC is through an impregnation method.^95–99^ Typically, the CC precursor solution and the PC are successively mixed and dried, followed by an annealing step to completely convert the precursors into active CC components. This strategy also enables homogeneous confinement of certain elements in small domains, thus allowing for facile manipulation of the metal composition. Although the majority of reported examples only describe a single type of metal, the deposition of multi-metallic oxide CCs has also been achieved. For example, RhCrO_*x*_ (a popular CC for photocatalytic water splitting) is commonly fabricated using this process.^100^ Tsuji *et al.* also performed a simple impregnation to load ternary-metallic Brownmillerite Ca_2_FeCoO_5_ NPs onto a TiO_2_ PC (Fig. 5d).^101^ However, this route requires high-temperature annealing to decompose the precursor salts, and this limits its application for thermally-unstable PCs. Moreover, fine-tuning of the CC size is difficult, and aggregation may adversely affect the catalytic performance of the CC.^102^ A solvothermal method can also be applied to grow metal (hydr)oxide or sulphide CCs directly on a PC.^103–107^ The straightforward addition of the PC into the EC synthesis solution can generate a CC/PC composite.

As mentioned in Section 2, a gas phase reaction can be employed to modify the composition of preformed oxide CCs. To promote the formation of high-quality chalcogenide, phosphide, nitride, and carbide CCs, which hardly form *via* impregnation or solvothermal methods, supplemental annealing treatments must be implemented. As a result, stable PCs with excellent thermal resistance (*e.g.*, C_3_N_4_) are mostly used for this approach.^108–111^

#### 3.1.3 Photodeposition

Irradiating the PC with light is a clever way to create an internal potential through the excitation of carriers, which can reduce or oxidize the precursor to form nanoparticulate CCs.^112^ Because photodeposition occurs at sites where photogenerated carriers preferentially migrate, CCs tend to nucleate on specific crystal planes of a PC, thus enabling location-controlled CC deposition.^14^ Photodeposition can also be performed at RT, and therefore, unstable materials [*e.g.*, (oxy)nitrides and (oxy)sulphides] can be used as the PC.^113,114^ Researchers have demonstrated that metal or metal oxide CCs, such as Rh, Pt, MnO_*x*_, and CoO_*x*_ can be loaded using this strategy.^115,116^ Recently, S- or P-containing precursors have been used for photodepositing metal sulphides and phosphates, respectively.^117–121^ Xu *et al.* photodeposited MoS_*x*_ and cobalt phosphate (CoPi) CCs on CdS nanowires using (NH_4_)_2_MoS_4_ and [Co(NO_3_)_2_ + Na_3_PO_4_], respectively (Fig. 5g),^122^ thereby validating the possibility of depositing complicated multi-metallic CCs on a PC under mild conditions.

In order to shed some light on the dependence of photocatalytic performance on the type of CC-loading method, apparent quantum yield (AQY) values (at 420 nm) for well-studied CdS/CC composites in the presence of hole scavengers are listed in Table 2. Although fair comparison between works under different conditions is difficult, rough tendency of AQY depending on loading method can be seen. CdS PCs loaded with CCs through physical mixing tend to show relatively lower AQY of around 10%. Meanwhile, CdS/CC composites assembled using hydrothermal and photodeposition methods can reach much higher AQY of up to ∼70%. These results suggest that the creation of an atomic-level PC/CC interface is paramount for a smooth carrier migration and reduced bulk recombination of photogenerated charge carriers in PCs.

### 3.2 Cocatalysts loading on photoelectrodes

In PEC systems, the photogenerated minority carriers diffuse to the semiconductor surface and the majority carriers are collected by the conductive substrate. For example, in photoanode containing n-type semiconductor, the electrons travel to the counter electrode, and the holes on photoanode surface and the electrons on the counter electrode surface are used to drive oxidation and reduction half-reactions, respectively. Therefore, unlike powdery PC systems, only one half-reaction (oxidation on n-type and reduction on p-type semiconductor) takes place on the PE surface, which requires the loading of appropriate CCs for each reaction.

Accordingly, efficient collection of majority carriers (holes in p-type and electrons in n-type semiconductors) requires a small resistance at the semiconductor/electrode interface and within the grain boundaries of semiconductor layer.^123^ Hence, CCs are typically loaded after fabricating high-quality PEs, wherein a mild CC-loading condition is necessary not to damage those prebuilt PEs. Impregnation by drop casting the CC precursor and a subsequent mild annealing is often applied when constructing CC/PE composites.^124–126^ Hydrothermal method can also be employed by immersing PEs in a reaction vessel containing CC precursors (Fig. 5e).^127–129^ These methods enable the formation of multi-metallic oxide CC layer on relatively unstable semiconductors like Ta_3_N_5_ (Table 3).^130^

An effective technique to homogeneously coat a CC layer on PEs is an electrodeposition. By applying a potential using a potentiostat, CCs can be deposited as reduced or oxidized species on the PE surface. Light-irradiation is sometimes applied during electrodeposition to assist adequate CC-loading under small applied potential. A representative example of an electrodeposited CC is a CoPi thin film, which is formed by oxidizing a Co precursor with a phosphate electrolyte.^131^ In addition, FeOOH, NiOOH, and CoOOH are popular OER CCs that can be anodically deposited in a similar way.^132–138^ These catalysts, coated as homogeneous thin films or NPs, have low absorption coefficients, which is a desirable feature for a CC. Considering that electrodeposition can be carried out at room temperature (RT), both robust BiVO_4_ and fragile Ta_3_N_5_ PCs can be used in this method (Fig. 5f).

### 3.3 Nanoparticle-adsorption approach

**Fig. 6:**
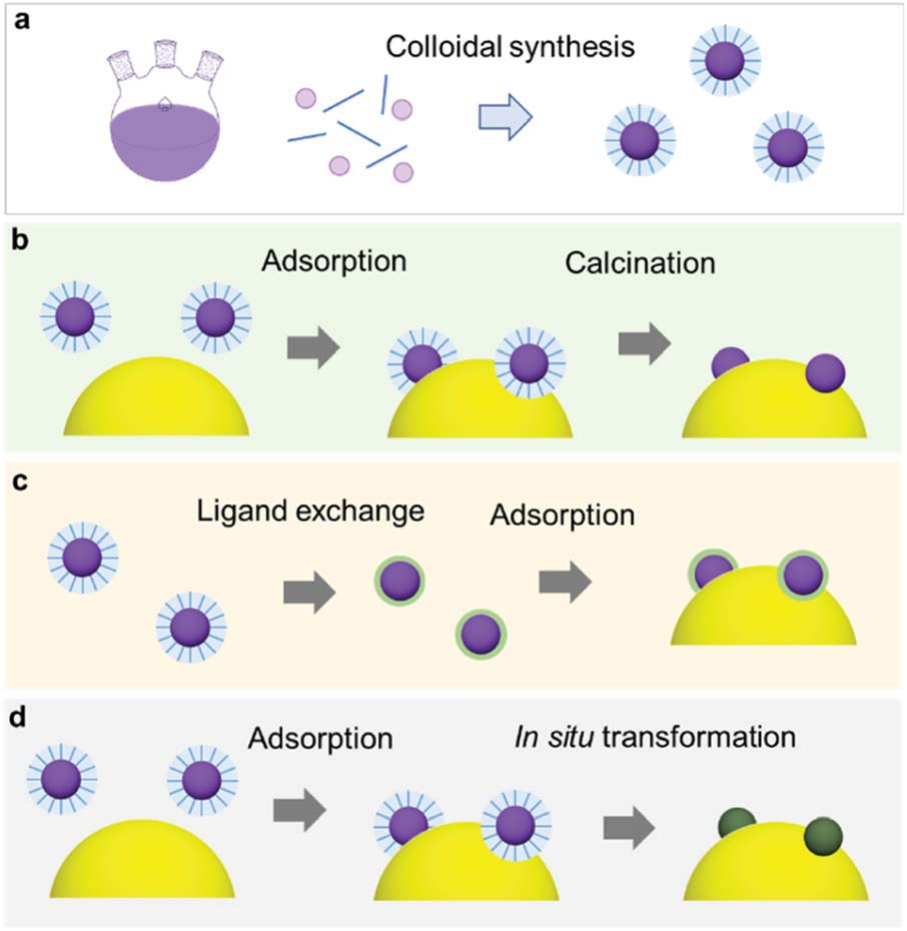
(a) Colloidal synthesis of monodisperse NPs; (b) NP loading *via* adsorption and subsequent calcination to remove protective organic ligands; (c) ligand exchange method to replace bulky organic ligands with small inorganic ions; (d) spontaneous ligand removal during catalysis.

**Fig. 7:**
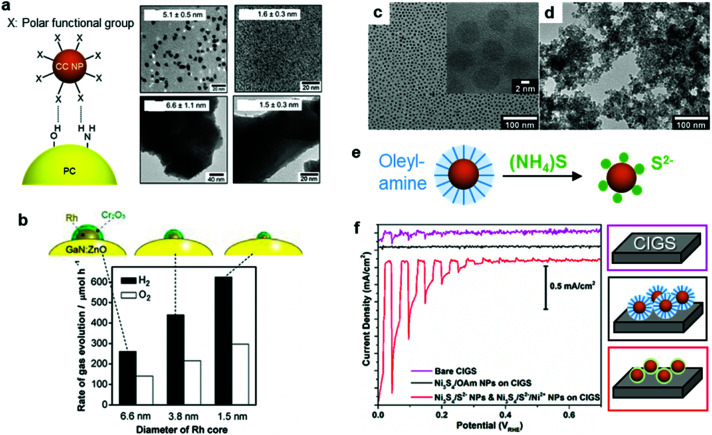
(a) Hydrogen-bonding mediated NP adsorption on PC and size-controlled PVP-coated Rh NPs (top) and Rh NP-loaded GaN:ZnO after calcination treatment (bottom); (b) photocatalytic activity of Cr_2_O_3_ shell-coated Rh NPs/GaN:ZnO; (c) oleylamine coated-Ni_3_S_4_ NPs; (d) S^2−^-stabilized Ni_3_S_4_ NPs obtained through (e) ligand exchange; (f) photocurrent scanning of bare, oleylamine coated-Ni_3_S_4_, and S^2−^-stabilized Ni_3_S_4_ NP-loaded CdS/Cu(In,Ga)Se_2_ PEs. Adapted with permission from (a and b) ref. 144, (c–f) ref. 147; copyright 2012 ACS and 2017 Wiley, respectively.

\Recent milestones in colloidal synthesis allow for the preparation of advanced NPs with controlled size and composition.^139^ Colloidal NPs represent ideal CC candidates because they can offer numerous active sites and enable uninterrupted light absorption by the semiconductor. Selective binding of organic ligands on the NP surface grants the opportunity to precisely engineer their surface.^140^ Specifically, organic molecules, which have polar functional groups at both ends, can link the NP and semiconductor through hydrogen bonding between the functional groups and OH units on the PC surface (Fig. 7a). However, such ligands behave as an insulating, carrier-blocking layer at the CC/semiconductor interface, and therefore, they should be eliminated (Fig. 6). The standard approach to remove these protective ligands and activate the NP catalysts involves applying an annealing treatment. Previous reports have shown that the required annealing temperature for complete ligand removal changes depending on the type of ligand and the PC support. Our group successfully deposited monodisperse Rh NPs as HER CCs on a GaN:ZnO PC.^141–145^ Specifically, ultrasmall polyvinylpyrrolidone (PVP) coated-Rh NPs were first adsorbed onto the PC using a linker molecule and then annealed in air at 400 °C (Fig. 7a). Interestingly, the size of the NPs remained ∼1 nm without aggregation after annealing. Negishi *et al.* also verified the crucial role of annealing to eliminate the ligands on Au clusters without changing their size.^146^ Such ultrafine NP deposition can only be realized through an NP-adsorption strategy. Depositing ultrasmall CCs can drastically enhance the photocatalytic performance of the composite because the NPs introduce a larger surface area and a greater number of active sites.^141^ However, larger NPs could reduce the number of cocatalytic sites at the same degree of loading, and this might shield the incident light needed for carrier generation. In this context, fine-tuning the NPs can contribute to a deeper fundamental understanding of the CC effect (*e.g.*, size and structure effects) on photocatalysis (Fig. 7b).^144^

Although a thermal treatment is favourable for ligand removal, elevated temperatures and a harsh environment may cause irreversible damage to the PC, PE and the CC NPs. Alternatively, replacing long-chain ligands with small surface-coordinating molecules is an effective way to increase the interfacial contact between the NPs and semiconductor under mild conditions. For example, our group treated oleylamine (OAm)-capped Ni_3_S_4_ NPs with S^2−^ at RT to replace the OAm and yield S^2−^-stabilized Ni_3_S_4_ NPs (Fig. 7c–e),^147^ which were then directly deposited on a CdS/Cu(In,Ga)Se_2_ PE through a layer-by-layer assembly process. Remarkably, the PE coated with S^2−^-Ni_3_S_4_ NPs exhibited an increase in cathodic photocurrent compared with the OAm-Ni_3_S_4_-NPs spin-coated PE, which highlighted the importance of establishing good contact between the CC and PE (Fig. 7f). Additionally, heating treatment to remove OAm from OAm-Ni_3_S_4_ layers at 300 °C resulted in deterioration of CdS/Cu(In,Ga)Se_2_ PE. These results demonstrate that the chemical ligand-removal process can enable the application of NP CCs to unstable PEs.

**Fig. 8:**
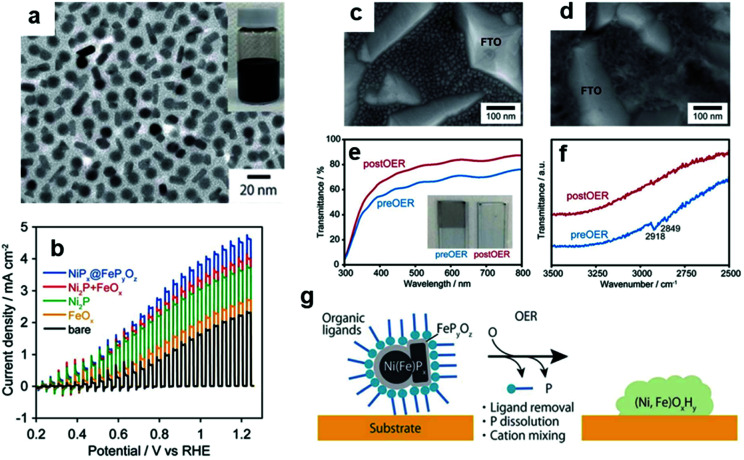
(a) TEM image of NiP_*x*_@FeP_*y*_O_*z*_ NPs; (b) photocurrent of BiVO_4_ PEs with/without loading NiP_*x*_@FeP_*y*_O_*z*_ NPs; SEM images of NiP_*x*_@FeP_*y*_O_*z*_ (c) before and (d) after CV on a fluorine-doped tin oxide (FTO) electrode; (e) transmittance spectra and (f) Fourier transform infrared (FTIR) spectra that confirm oleylamine ligand removal from NiP_*x*_@FeP_*y*_O_*z*_ NPs; (g) schematic diagram illustrating the transformation of NPs on BiVO_4_. Adapted with permission from ref. 148; copyright 2018 RSC.

By exploiting the transformation phenomenon of ECs during catalysis, we demonstrated that applying potential for OER to a NiP_*x*_@FeP_*y*_O_*z*_ NP-loaded electrode transformed the NPs into highly OER active NiFeOOH film while simultaneously removing their organic ligands (Fig. 8).^148^ Notably, the colour of the NPs changed from black (phosphide) to colourless (hydroxide), with enhanced transparency for incident visible light. Such a characteristic transformation is highly advantageous for coupling NPs with PCs and PEs under mild conditions. Evidently, the NiP_*x*_@FeP_*y*_O_*z*_ NP deposition substantially increased the photocurrent of the BiVO_4_ PE without any post-treatment (Fig. 8c–g), showing that homogeneous NP dispersion can be used as ready-to-use CC ink.

## 4. Application of ECs to CCs

As discussed in Section 3, PCs and PEs can be modified with CCs through a variety of strategies to significantly enhance their water-splitting efficiencies. Potential opportunities to further improve the activity could be foreseen if the factors limiting the activity could be identified. For example, what are the core principles relevant for unlocking high-performance CC/PC and CC/PE systems and bypassing rigorous material screening experiments? In this section, we introduce fundamental concepts that play a significant role in designing advanced ECs as CCs for photocatalytic and PEC water splitting.

### 4.1 Energy level matching at the CC/semiconductor interface

**Fig. 9:**
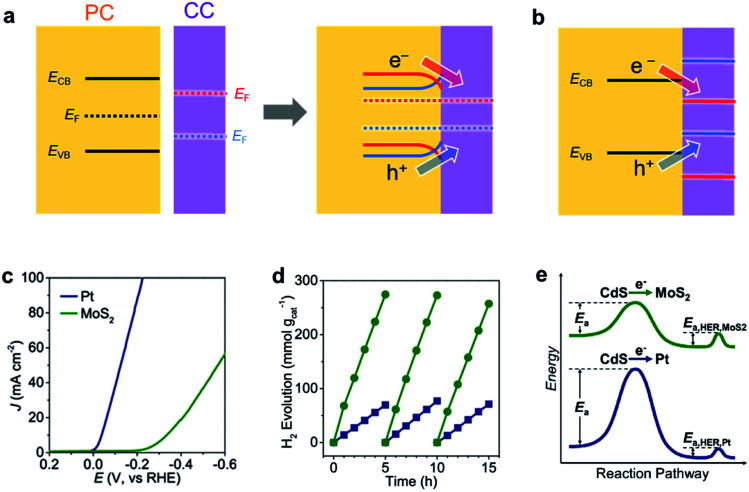
Energy level matching of a PC semiconductor with a CC for (a) metal CC and (b) semiconductor CC [red and blue cases create ohmic contact (Schottky barrier) for photogenerated electrons (holes) and holes (electrons), respectively]; (c) CV comparison of electrochemical HER activity of Pt and MoS_2_ ECs in 0.5 M H_2_SO_4_; (d) photocatalytic HER activity of Pt/CdS and MoS_2_/CdS composites under visible-light (>400 nm) irradiation; (e) schematic reaction profile showing the activation energy from light absorption to H_2_ evolution. Adapted with permission from (c–e) ref. 153; copyright 2019 ACS.

When an EC comes in contact with a semiconductor, the band structure of the semiconductor is modulated.^149^ In general, an electronic contact between a metallic CC and a semiconductor initiates an electron transfer between the semiconductor and the CC until their Fermi levels (*E*_F_; the energy level with a 50% probability of electron occupation) equilibrate, which forms a space charge layer. In the space charge layer, the band edge energies of the semiconductor are continuously shifted by the electric field between the semiconductor and the metallic CC. As a result, the energy bands of the semiconductor bend toward the CC. The band bends upward (downward) when the *E*_F_ of PC is higher (lower) than the *E*_F_ of CC. Upward band bending creates an energetic upslope (Schottky barrier) for electron transfer but facilitates hole transfer from PC to CC, which encourages photo–oxidation reaction on CCs.^149,152^ On the contrary, downward band bending promotes electron transfer (ohmic contact) but hinders hole transfer from PC to CC, which is desirable for photo–reduction reaction. Therefore, proper energy level matching to minimize the barrier height or create an ohmic contact for specific carriers is vital for a maximum utilization of photogenerated carriers. Although nanosized CCs have limited capabilities for donating or accepting electrons, loading sufficient amount of CC NPs can still cause band bending in the semiconductor, which has been verified experimentally using Kelvin probe force microscopy and surface photovoltage measurements.^150,151^ For a CC with semiconductor-like properties, its band alignment with the PC and PE should also be considered to assess the charge transfer efficiency at their interface (Fig. 9b).^149^

Chen *et al.* clearly demonstrated the beneficial effect of an appropriate CC/PC junction on the photocatalytic activity by loading Pt or MoS_2_ CCs on the surface of CdS PCs.^153^ Although Pt has a higher HER activity than MoS_2_ as an EC (Fig. 9c), the photocatalytic HER activity of the MoS_2_-loaded CdS was superior to that of the Pt-loaded CdS (Fig. 9d). This observation arose from the differences in their electron transfer rates. Specifically, the deeper *E*_F_ of Pt caused a greater upward band bending effect in CdS, which simultaneously disturbed the photoexcited electron transfer from CdS to Pt. This suggests that a lower activation potential for electron transfer from CdS makes MoS_2_ more suitable as a CC than Pt (Fig. 9e). Therefore, the relative performances of ECs do not necessarily reflect their relative applicability as CCs.

To evaluate the carrier transfer efficiency at CC/semiconductor junctions, key parameters (*e.g.*, work function of the metal CC, *E*_F_ of the semiconductor) deserve deeper investigations. In the case of metals, standard work function data can be used to estimate the bending direction and Schottky barrier height at CC/PC junctions; in contrast, *E*_F_ and bandgap values for complex heterogeneous CCs are not readily available, which makes evaluations of their band alignment difficult. Nevertheless, DFT and first-principle calculations can provide critical insights on the fundamental electronic structure of EC materials (including band structure, density of states and *E*_F_), enabling rapid prediction of suitable PC/CC combinations.

**Fig. 10:**
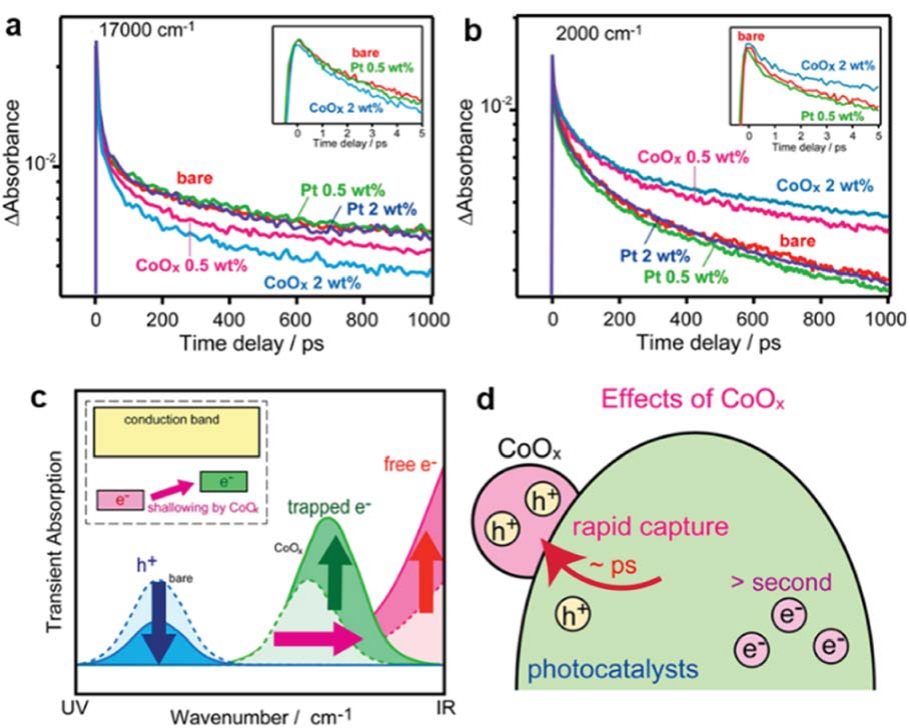
Decay of transient absorption at (a) 17 000 cm^−1^ and (b) 2000 cm^−1^ for bare, CoO_*x*_-loaded, and Pt-loaded LaTiO_2_N after excited by 500 nm laser pulses; (c and d) schematics showing the change in carrier dynamics upon CoO_*x*_ CC loading. Adapted with permission from ref. 157; copyright 2014 ACS.

Even if the interfacial band alignments are unknown, the interfacial carrier dynamics can be measured through optical experiments, which can be used to assess the PC/CC interface.^154^ Transient absorption spectroscopy (TAS) monitors the photogenerated carrier dynamics in a PC by recording the temporal absorption evolution after a pulse excitation.^155^ For example, TAS reveals (i) how electrons at the conduction band minimum behave in a PC/CC composite after photoexcitation and (ii) the subsequent ultrafast intraband relaxation of hot electrons by probing the absorption in the infrared region derived from free electrons.^155,156^ Although TAS has been conducted on clear quantum-dot dispersions, TAS measurements for solid-state bulk materials in reflectance mode are still uncommon. Yamakata *et al.* showed how Pt and CoO_*x*_ CCs affected the carrier dynamics when deposited on an LaTiO_2_N PC in the solid state.^157^ Upon CoO_*x*_ loading, visible (17 000 cm^−1^) and IR (2000 cm^−1^) probes traced a temporal decrease and increase in the population of holes and free electrons in LaTiO_2_N, respectively, after a 500 nm laser pulse excitation (Fig. 10). These results indicated that CoO_*x*_ can rapidly extract photogenerated holes in LaTiO_2_N to dramatically extend the lifetime of electrons. In contrast, Pt could not effectively extract photogenerated electrons because of the trapping mechanism occurring at mid-gap states in the LaTiO_2_N. These TAS findings help pinpoint which specific process is the bottleneck in attaining excellent activity. Therefore, applying ultrafast spectroscopy to CC/PC(PE) systems could be very useful in providing guidelines for designing CC materials.

### 4.2 Assessment of CC activity

**Fig. 11:**
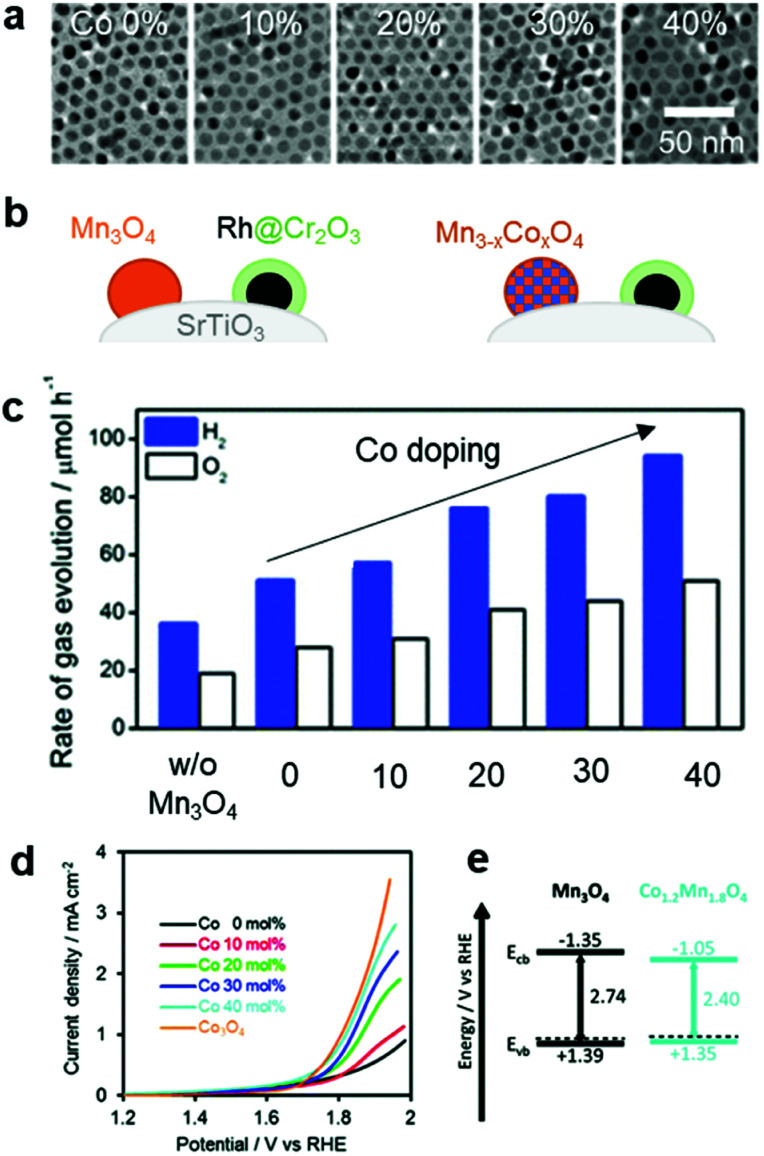
Co-doping effect on the overall water-splitting activity of SrTiO_3_: (a) TEM images of Mn_3_O_4_ doped with various Co concentrations; (b) schematic of CC-loaded photocatalyst; (c) photocatalytic trend as a function of Co dopant concentration in Mn_3_O_4_ CCs; (d) electrochemical OER activities of Co_*x*_Mn_3−*x*_O_4_ NPs in a neutral electrolyte (0.1 M PBS); (e) calculated band structures of Mn_3_O_4_ and Co_*x*_Mn_3−*x*_O_4_ NPs. Adapted with permission from ref. 145; copyright 2018 RSC.

In addition to the interfacial carrier dynamics, the intrinsic electrocatalytic activity of the CC material is still a significant descriptor of photocatalytic and PEC performance. Since the CCs serve as highly-active sites to drive redox reactions (like ECs), an understanding and evaluation of their intrinsic HER and OER activities are requisite for enhancing the catalytic performance. Notably, their catalytic activities can be measured with a CC-loaded electrode using an electrochemical system. In EC studies, the catalytic activities are typically monitored under strong acidic or alkaline conditions, which are advantageous in showing their maximum activities. However, such extreme pH conditions cannot be applied in photocatalytic and PEC systems because they would likely cause chemical damage to the semiconductor.^158^ Since the stability of the photoactive semiconductor is the top priority for water splitting, it is important to conduct assessments at a practically-applicable pH. Even if an EC exhibits excellent performance under specific pH conditions, that same material might not be equally effective as CCs under different pH conditions.^159^ Therefore, it is necessary to examine the CC performance in an environment similar to that optimized for the photocatalytic and PEC systems. For example, based on the synergistic improvement of OER activity by a bimetallic Co–Mn oxide EC,^160^ our group investigated the effect of Co doping on the catalytic OER activity of Mn_3_O_4_ NPs on the SrTiO_3_ PC, and found that increasing the Co doping content improved the water-splitting activity (Fig. 11).^145^ By measuring the OER activity of a Co_*x*_Mn_3−*x*_O_4_ NP-loaded electrode in neutral pH [0.1 M phosphate buffer solution (PBS)], we found that the Co content monotonically enhanced the OER activity (Fig. 11d). The calculated band structure of the Co_*x*_Mn_3−*x*_O_4_ NPs had similar valence band levels, regardless of the Co content, which indicated that the enhanced OER kinetics achieved by the Co-doping in Mn_3_O_4_ CC primarily contributed to boosting the overall photocatalytic water-splitting activity by accelerating slow OER process (Fig. 11e). Electrochemical assessments under similar conditions as those used for the photocatalytic and PEC systems can offer more accurate predictions of an EC's suitability as a CC. Recently reported ECs that are active over universal pH represent promising candidates as CCs in various photocatalytic and PEC environments.^161–163^

### 4.3 Prevention of backward reaction

**Fig. 12:**
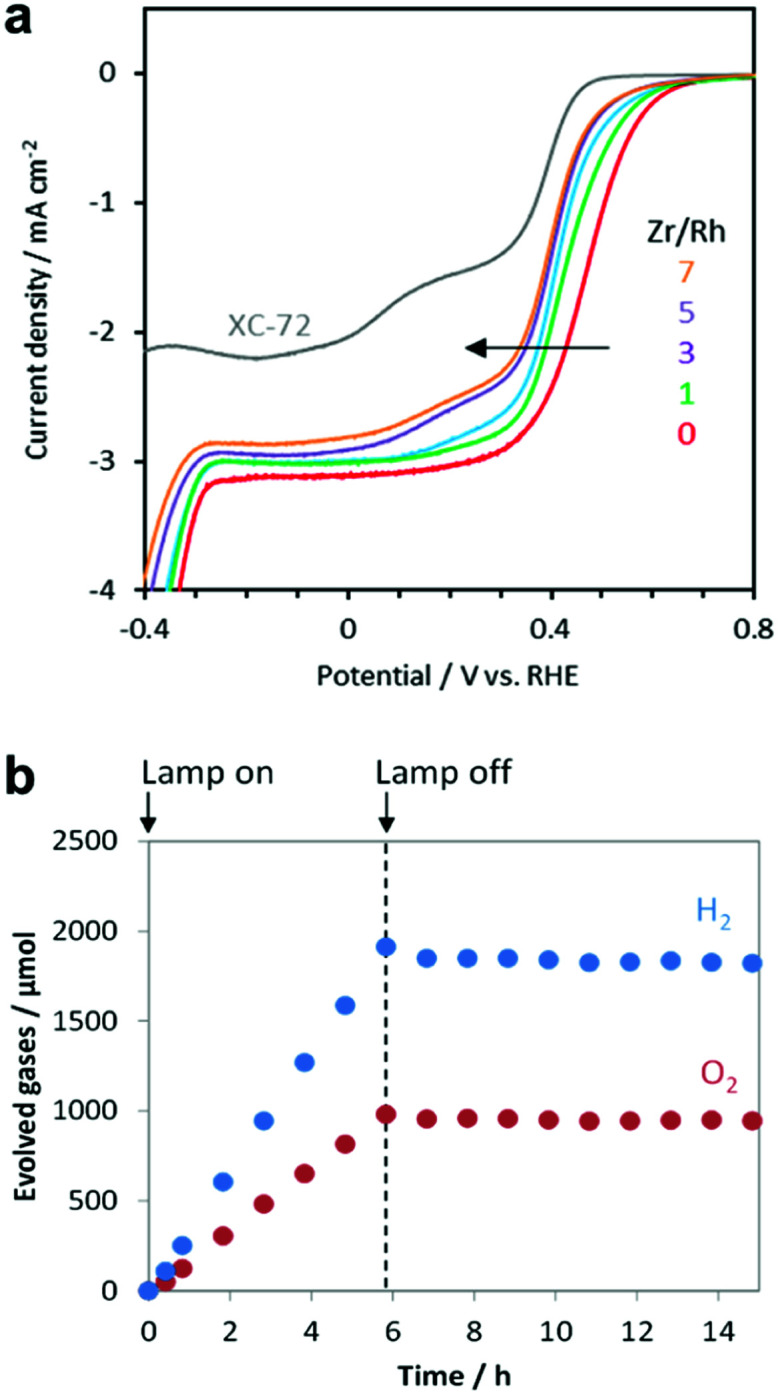
(a) Current–potential curves of RhZrO_*x*_ (Zr/Rh: 0–7 w/w)-loaded carbon supports in O_2_-saturated 1 M Na_2_SO_4_ (pH 7); (b) gas evolution over time by RhZrO_*x*_/SrTiO_3_:Al (Rh = 0.1 wt% and Zr = 0.5 wt% *vs.* SrTiO_3_ : Al) during and after light irradiation (300 W Xe lamp). Adapted with permission from ref. 169; copyright 2020 RSC.

The oxygen reduction reaction (ORR) is usually not an issue to consider for electrochemical systems because they employ a divided cell configuration, wherein the HER and OER electrodes are placed in separated cells. However, the ORR must be considered in overall water splitting using powdered PCs because both half-reactions take place in a single reactor. Outstanding HER CCs, such as Pt and Rh metals, tend to actively catalyse the ORR,^22^ which consumes the H_2_ and O_2_ evolved during the photocatalysis, thus reducing the H_2_ uptake.^164^ Strategies to effectively suppress this backward reaction and increase the quantity of evolved H_2_ include the application of protective coatings on the CCs (*e.g.*, amorphous oxides such as CrO_*x*_,^165^ TaO_*x*_,^166^ SiO_*x*_,^167^ and ZrO_*x*_ (ref. 168 and 169)). Our electrochemical measurements demonstrated that the ZrO_*x*_ matrix could suppress the ORR of Rh^3+^ active sites, which confirmed the O_2_-blocking ability of ZrO_*x*_ (Fig. 12a) and prevented the loss of H_2_-uptake in photocatalytic overall water splitting by ZrRhO_*x*_/SrTiO_3_:Al (Fig. 12b). Evaluating the ORR activities of CC materials in addition to their HER and OER activities can provide insights relevant for improving the total photocatalytic activity.

### 4.4 Structural and chemical transformations of CCs during photocatalysis

**Fig. 13:**
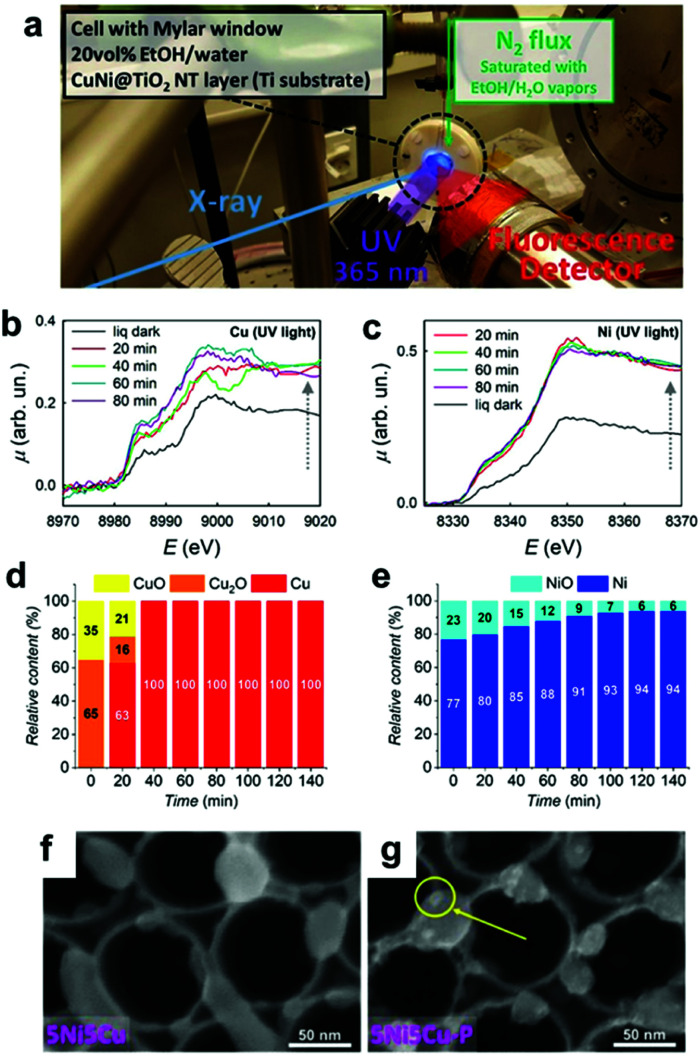
(a) *Operando* XAS experimental setup; (b) Cu K-edge and (c) Ni K-edge XAS spectra of 5Ni5Cu–TiO_2_ in a water−ethanol under UV irradiation for various exposure times; (d) Cu and (e) Ni phase contents determined by *operando* XAS for 5Ni5Cu–TiO_2_; SEM images of 5Ni5Cu–TiO_2_ (f) before and (g) after photocatalysis (a yellow circle indicates metallic NPs deposited under the UV irradiation). Adapted with permission from ref. 171; copyright 2020 ACS.

Similar to ECs, nanosized CCs can undergo structural and chemical transformations during photocatalytic and PEC water-splitting. In fact, our group's study revealed that the NiP_*x*_@FeP_*y*_O_*z*_ CCs on BiVO_4_ PE transformed into NiFeOOH based on post-OER characterizations with X-ray photoelectron spectroscopy (XPS) and Fourier transform infrared spectroscopy (FTIR).^148^ We also observed a compositional change in the novel Rh–Cl–Zr–O CCs deposited on SrTiO_3_:Al PCs under operating conditions. After the photocatalysis, XPS analyses detected the removal of Cl from the initial Rh–Cl–Zr–O CC to form RhZrO_*x*_ during photocatalysis.^169^ These results indicated the importance of post-catalysis characterization in determining the actual active species. Additionally, *in situ*/*operando* characterization techniques are powerful tools for monitoring the temporal evolution of CCs under the relevant working conditions, and they can help us to deeply understand the transformation mechanism of CCs.^170^ Recently, synchrotron-based characterizations have provided valuable data with high temporal resolution, even for a small concentration of CCs. Altomare and co-workers conducted *operando* X-ray absorption spectroscopy (XAS) experiments for CuNi CCs loaded onto TiO_2_ during photocatalysis (Fig. 13a).^171^ At the early stage of the reaction, the preformed CuO_*x*_ and NiO species were reduced to metallic Cu and Ni, thus forming CuNi alloy CCs (Fig. 13b–e). Subsequently, the H_2_ evolution rate increased as the transformation progressed, which identified the metallic CuNi as the actual active sites. *Operando* XAS can also be used to confirm the stability of CCs in a sustained catalytic setting. Fundamentally, the integrated utilization of the methods discussed here is highly desirable to accelerate progress in designing highly-active CC materials.

## 5. Conclusions and outlook

In this perspective, we have discussed recent developments in EC materials and their application as CCs in photocatalytic and PEC water-splitting systems. Although significant efforts have already been directed toward the construction of high-performance CC/PC and CC/PEC composites, exploration of CC materials still lags its EC counterpart. If methodologies to properly hybridize active EC materials with a variety of photoactive semiconductors (including unstable chalcogenides and nitrides) are developed, the accumulated knowledge regarding ECs will dramatically accelerate advances in photocatalytic and PEC systems. We believe that the colloidal approach using NPs of active EC materials with controllable sizes and compositions represents a promising solution to bridge the studies of ECs and CCs.

However, there are urgent issues that must be overcome in order to further enhance their photocatalytic performance. For particulate PC systems, spatial separation of HER and OER sites is highly desirable to efficiently promote both half-reactions. Facet-selective photodeposition of HER and OER CCs has been realized on PCs (*e.g.*, SrTiO_3_:Al and BiVO_4_) by utilizing the intrinsic built-in potential generated from the work function difference between the PC crystal planes.^76,115^ To realize such site-specific CC deposition in other loading methods, exploiting the preferential adsorption of surfactants on different crystal planes of PCs could be useful.^172^ The surfactants on CC precursor complex or NP surface have functional groups that could interact with coordinatively unsaturated atoms on the PC surface *via* dynamic adsorption and desorption. Again, the distinct atomic arrangement on the crystal facets of PCs might affect the binding affinities of incoming functional groups. For example, the (111) surface of Cu_2_O PCs contains more Cu dangling bonds that can be passivated by polar functional ligands.^173^ In turn, CCs could be preferentially loaded on the exposed (100) surface of Cu_2_O through proper linker molecules. Likewise, PtCl_6_^2−^ interacted strongly with OH groups on the anatase TiO_2_ (101) surface, whereas Pt(NH_3_)_4_^2+^ does not.^174^ As such, this strategy can only be implemented on limited PCs and CCs, thus expanding the library of applicable materials is of primary importance to improve the poor STH efficiencies in PC systems.

Another crucial factor that needs to be taken into account is the formation of an intimate interfacial contact between CCs and semiconductors. Although CCs are usually loaded on as-prepared PC powder and PEs, the presence of pre-existing defective and/or insulative oxide layers on the semiconductor surface might cause charge recombination and/or carrier transfer blocking, and therefore should be removed before CC deposition.^175^ Acid treatment is a simple and effective way to dissolve such surface oxide layers.^175^

Ensuring long-term durability of CCs is also critical in maintaining excellent photocatalytic performance over extended operation periods. Since small amount of CCs is usually loaded on NPs or thin films, CC degradation can potentially occur during photocatalysis through dissolution and physical removal.^125^ The loss of carrier extraction capability of CCs could lead to self-corrosion of PCs due to uncompensated total charge.^19^ Employing newly developed efficient and durable multi-metallic compound EC materials as CCs is a straightforward but promising way in accelerating the discovery of high-performance PC/CC combinations. Aside from improving the chemical and physical durability, CC regeneration by feeding CC precursor during operation could be a logical solution in prolonging the lifetime of practical photocatalytic systems.^176^

As stated from Section 3, the need to control the CC morphology, internal electronic structure, and intrinsic surface catalytic activity, in addition to the CC's interfacial structure with the semiconductors, make advancements in this field challenging. However, recent advances in characterization techniques such as ultrafast spectroscopy and synchrotron-based *in situ* analyses have gradually revealed the crucial role of such factors in determining the bottlenecks of photocatalytic systems. In addition to developing these technologies, acquiring fundamental properties from basic electrocatalytic analysis and theoretical DFT and *ab initio* calculation can potentially contribute to the understanding and construction of a sustainable and highly-efficient solar-driven photocatalytic water-splitting system.

## Author contributions

MS conceived the topic and structure of the work. All the authors contributed to the discussion, writing, editing, and revision of this work.

## Conflicts of interest

There are no conflicts to declare.

## Supplementary Material

## References

[cit1] Wigley T. M. L. (2017). Clim. Chang..

[cit2] Peter S. C. (2018). ACS Energy Lett.

[cit3] Sánchez-Bastardo N., Schlögl R., Ruland H. (2021). Ind. Eng. Chem. Res..

[cit4] Nonobe Y. (2017). IEEJ Trans. Electr. Electron. Eng..

[cit5] Ashik U. P. M., Wan Daud W. M. A., Abbas H. F. (2015). Renew. Sustain. Energy Rev..

[cit6] Dutta S. (2021). Energy Fuels.

[cit7] Shaner M. R., Atwater H. A., Lewis N. S., McFarland E. W. (2016). Energy Environ. Sci..

[cit8] Jia J., Seitz L. C., Benck J. D., Huo Y., Chen Y., Ng J. W. D., Bilir T., Harris J. S., Jaramillo T. F. (2016). Nat. Commun..

[cit9] Wang S., Lu A., Zhong C.-J. (2021). Nano Convergence.

[cit10] Wang Q., Domen K. (2019). Chem. Rev..

[cit11] Kang D., Kim T. W., Kubota S. R., Cardiel A. C., Cha H. G., Choi K.-S. (2015). Chem. Rev..

[cit12] Hisatomi T., Domen K. (2019). Nat. Catal..

[cit13] Nishiyama H., Yamada T., Nakabayashi M., Maehara Y., Yamaguchi M., Kuromiya Y., Tokudome H., Akiyama S., Watanabe T., Narushima R., Okunaka S., Shibata N., Takata T., Hisatomi T., Domen K. (2021). Nature.

[cit14] Jiang C., Moniz S. J. A., Wang A., Zhang T., Tang J. (2017). Chem. Soc. Rev..

[cit15] Wu H., Tan H. L., Toe C. Y., Scott J., Wang L., Amal R., Ng Y. H. (2020). Adv. Mater..

[cit16] Higashi T., Nishiyama H., Suzuki Y., Sasaki Y., Hisatomi T., Katayama M., Minegishi T., Seki K., Yamada T., Domen K. (2019). Angew. Chem., Int. Ed..

[cit17] Yang J., Wang D., Han H., Li C. (2013). Acc. Chem. Res..

[cit18] Takanabe K. (2017). ACS Catal..

[cit19] Ning X., Lu G. (2020). Nanoscale.

[cit20] Ran J., Zhang J., Yu J., Jaroniec M., Qiao S. Z. (2014). Chem. Soc. Rev..

[cit21] Qiao W., Tao H. B., Liu B., Chen J. (2019). Small.

[cit22] Seh Z. W., Kibsgaard J., Dickens C. F., Chorkendorff I., Nørskov J. K., Jaramillo T. F. (2017). Science.

[cit23] Zhu J., Hu L., Zhao P., Lee L. Y. S., Wong K. Y. (2020). Chem. Rev..

[cit24] Song J., Wei C., Huang Z.-F., Liu C., Zeng L., Wang X., Xu Z. J. (2020). Chem. Soc. Rev..

[cit25] Nørskov J. K., Bligaard T., Logadottir A., Kitchin J. R., Chen J. G., Pandelov S., Stimming U. (2005). J. Electrochem. Soc..

[cit26] Jaramillo T. F., Jørgensen K. P., Bonde J., Nielsen J. H., Horch S., Chorkendorff I. (2007). Science.

[cit27] Liu P., Zhu J., Zhang J., Xi P., Tao K., Gao D., Xue D. (2017). ACS Energy Lett.

[cit28] Chen Y., Yang K., Jiang B., Li J., Zeng M., Fu L. (2017). J. Mater. Chem. A.

[cit29] El-Refaei S. M., Russo P. A., Pinna N. (2021). ACS Appl. Mater. Interfaces.

[cit30] Jiang Y., Lu Y. (2020). Nanoscale.

[cit31] Kuang M., Huang W., Hegde C., Fang W., Tan X., Liu C., Ma J., Yan Q. (2020). Mater. Horizons.

[cit32] Fu G., Lee J.-M. (2019). J. Mater. Chem. A.

[cit33] Tang C., Gan L., Zhang R., Lu W., Jiang X., Asiri A. M., Sun X., Wang J., Chen L. (2016). Nano Lett..

[cit34] Kwak I. H., Kwon I. S., Debela T. T., Abbas H. G., Park Y. C., Seo J., Ahn J.-P., Lee J. H., Park J., Kang H. S. (2020). ACS Nano.

[cit35] Rezaie A. A., Lee E., Luong D., Yapo J. A., Fokwa B. P. T. (2021). ACS Mater. Lett..

[cit36] Park S. H., Jo T. H., Lee M. H., Kawashima K., Mullins C. B., Lim H.-K., Youn D. H. (2021). J. Mater. Chem. A.

[cit37] Zheng X., Chen Y., Bao X., Mao S., Fan R., Wang Y. (2020). ACS Catal..

[cit38] Zhao S., Berry-Gair J., Li W., Guan G., Yang M., Li J., Lai F., Corà F., Holt K., Brett D. J. L., He G., Parkin I. P. (2020). Adv. Sci..

[cit39] Naito T., Shinagawa T., Nishimoto T., Takanabe K. (2021). Inorg. Chem. Front..

[cit40] Over H. (2021). ACS Catal..

[cit41] Bodhankar P. M., Sarawade P. B., Singh G., Vinu A., Dhawale D. S. (2021). J. Mater. Chem. A.

[cit42] Pascuzzi M. E. C., Man A. J. W., Goryachev A., Hofmann J. P., Hensen E. J. M. (2020). Catal. Sci. Technol..

[cit43] Man I. C., Su H.-Y., Calle-Vallejo F., Hansen H. A., Martínez J. I., Inoglu N. G., Kitchin J., Jaramillo T. F., Nørskov J. K., Rossmeisl J. (2011). ChemCatChem.

[cit44] Zhang B., Zheng X., Voznyy O., Comin R., Bajdich M., García-Melchor M., Han L., Xu J., Liu M., Zheng L., de Arquer F. P. G., Dinh C. T., Fan F., Yuan M., Yassitepe E., Chen N., Regier T., Liu P., Li Y., De Luna P., Janmohamed A., Xin H. L., Yang H., Vojvodic A., Sargent E. H. (2016). Science.

[cit45] Kim S., Mizuno H., Saruyama M., Sakamoto M., Haruta M., Kurata H., Yamada T., Domen K., Teranishi T. (2020). Chem. Sci..

[cit46] Anantharaj S., Ede S. R., Sakthikumar K., Karthick K., Mishra S., Kundu S. (2016). ACS Catal..

[cit47] Zhang T., Zhu Y., Lee J. Y. (2018). J. Mater. Chem. A.

[cit48] Shang X., Tang J.-H., Dong B., Sun Y. (2020). Sustain. Energy Fuels.

[cit49] Zhang G., Li Y., Xiao X., Shan Y., Bai Y., Xue H.-G., Pang H., Tian Z., Xu Q. (2021). Nano Lett..

[cit50] Guan D., Ryu G., Hu Z., Zhou J., Dong C.-L., Huang Y.-C., Zhang K., Zhong Y., Komarek A. C., Zhu M., Wu X., Pao C.-W., Chang C.-K., Lin H.-J., Chen C.-T., Zhou W., Shao Z. (2020). Nat. Commun..

[cit51] Cui M., Yang C., Li B., Dong Q., Wu M., Hwang S., Xie H., Wang X., Wang G., Hu L. (2021). Adv. Energy Mater..

[cit52] Dionigi F., Strasser P. (2016). Adv. Energy Mater..

[cit53] Liu D., Ai H., Li J., Fang M., Chen M., Liu D., Du X., Zhou P., Li F., Lo K. H., Tang Y., Chen S., Wang L., Xing G., Pan H. (2020). Adv. Energy Mater..

[cit54] Zu M. Y., Wang C., Zhang L., Zheng L. R., Yang H. G. (2019). Mater. Horizons.

[cit55] Zhu Y., Zhou W., Zhong Y., Bu Y., Chen X., Zhong Q., Liu M., Shao Z. (2017). Adv. Energy Mater..

[cit56] Wu D., Kusada K., Yoshioka S., Yamamoto T., Toriyama T., Matsumura S., Chen Y., Seo O., Kim J., Song C., Hiroi S., Sakata O., Ina T., Kawaguchi S., Kubota Y., Kobayashi H., Kitagawa H. (2021). Nat. Commun..

[cit57] Zhang J., Wang T., Pohl D., Rellinghaus B., Dong R., Liu S., Zhuang X., Feng X. (2016). Angew. Chem., Int. Ed..

[cit58] Xu H., Wei J., Zhang K., Shiraishi Y., Du Y. (2018). ACS Appl. Mater. Interfaces.

[cit59] Zhu C., Yin Z., Lai W., Sun Y., Liu L., Zhang X., Chen Y., Chou S. L. (2018). Adv. Energy Mater..

[cit60] Jiang J., Liu Q., Zeng C., Ai L. (2017). J. Mater. Chem. A.

[cit61] Xiong B., Chen L., Shi J. (2018). ACS Catal..

[cit62] McCrory C. C. L., Jung S., Peters J. C., Jaramillo T. F. (2013). J. Am. Chem. Soc..

[cit63] Wang M., Zhang L., He Y., Zhu H. (2021). J. Mater. Chem. A.

[cit64] Garsany Y., Baturina O. A., Swider-Lyons K. E., Kocha S. S. (2010). Anal. Chem..

[cit65] Chaudhari N. K., Jin H., Kim B., Lee K. (2017). Nanoscale.

[cit66] Garsany Y., Baturina O. A., Swider-Lyons K. E., Kocha S. S. (2010). Anal. Chem..

[cit67] Zhu C., Shi Q., Feng S., Du D., Lin Y. (2018). ACS Energy Lett.

[cit68] Stevens M. B., Enman L. J., Batchellor A. S., Cosby M. R., Vise A. E., Trang C. D. M., Boettcher S. W. (2016). Chem. Mater..

[cit69] Jin S. (2017). ACS Energy Lett.

[cit70] Li W., Xiong D., Gao X., Liu L. (2019). Chem. Commun..

[cit71] Chen P., Xu K., Fang Z., Tong Y., Wu J., Lu X., Peng X., Ding H., Wu C., Xie Y. (2015). Angew. Chem., Int. Ed..

[cit72] Cai W., Chen R., Yang H., Tao H. B., Wang H.-Y., Gao J., Liu W., Liu S., Hung S.-F., Liu B. (2020). Nano Lett..

[cit73] Xiao J., Vequizo J. J. M., Hisatomi T., Rabeah J., Nakabayashi M., Wang Z., Xiao Q., Li H., Pan Z., Krause M., Yin N., Smith G., Shibata N., Brückner A., Yamakata A., Takata T., Domen K. (2021). J. Am. Chem. Soc..

[cit74] Bai S., Yin W., Wang L., Li Z., Xiong Y. (2016). RSC Adv..

[cit75] Hosogi Y., Shimodaira Y., Kato H., Kobayashi H., Kudo A. (2008). Chem. Mater..

[cit76] Takata T., Jiang J., Sakata Y., Nakabayashi M., Shibata N., Nandal V., Seki K., Hisatomi T., Domen K. (2020). Nature.

[cit77] Zhao J., Fu B., Li X., Ge Z., Ma B., Chen Y. (2020). ACS Appl. Energy Mater..

[cit78] Ren D., Liang Z. Z., Ng Y. H., Zhang P., Xiang Q., Li X. (2020). Chem. Eng. J..

[cit79] Lu X., Xie J., Liu S., Adamski A., Chen X., Li X. (2018). ACS Sustain. Chem. Eng..

[cit80] Tian L., Min S., Wang F., Zhang Z. (2019). J. Phys. Chem. C.

[cit81] Ren D., Liang Z. Z., Ng Y. H., Zhang P., Xiang Q., Li X. (2020). Chem. Eng. J..

[cit82] Zhang J., Yao W., Huang C., Shi P., Xu Q. (2017). J. Mater. Chem. A.

[cit83] Qin Z., Chen Y., Huang Z., Su J., Guo L. (2017). J. Mater. Chem. A.

[cit84] Cheng C., Zong S., Shi J., Xue F., Zhang Y., Guan X., Zheng B., Deng J., Guo L. (2020). Appl. Catal. B Environ..

[cit85] Zhong W., Tu W., Feng S., Xu A. (2019). J. Alloys Compd..

[cit86] Irfan R. M., Tahir M. H., Iqbal S., Nadeem M., Bashir T., Maqsood M., Zhao J., Gao L. (2021). J. Mater. Chem. C.

[cit87] Shen Z.-K., Yuan Y.-J., Wang P., Bai W., Pei L., Wu S., Yu Z.-T., Zou Z. (2020). ACS Appl. Mater. Interfaces.

[cit88] Du H., Guo H.-L., Liu Y.-N., Xie X., Liang K., Zhou X., Wang X., Xu A.-W. (2016). ACS Appl. Mater. Interfaces.

[cit89] Liu Y., Wang B., Zhang Q., Yang S., Li Y., Zuo J., Wang H., Peng F. (2020). Green Chem..

[cit90] Sun X., Du H. (2019). ACS Sustain. Chem. Eng..

[cit91] Chang K., Mei Z., Wang T., Kang Q., Ouyang S., Ye J. (2014). ACS Nano.

[cit92] Liu Y., Wang B., Zhang Q., Yang S., Li Y., Zuo J., Wang H., Peng F. (2020). Green Chem..

[cit93] Chao Y., Zheng J., Zhang H., Ma Y., Li F., Tan Y., Zhu Z. (2018). Energy Technol..

[cit94] Ma B., Liu Y., Li J., Lin K., Liu W., Zhan H. (2016). Int. J. Hydrogen Energy.

[cit95] Liang X., Xie J., Xiong J., Gong L., Li C. M. (2018). Sustain. Energy Fuels.

[cit96] Pihosh Y., Nandal V., Minegishi T., Katayama M., Yamada T., Seki K., Sugiyama M., Domen K. (2020). ACS Energy Lett.

[cit97] Zong X., Han J., Ma G., Yan H., Wu G., Li C. (2011). J. Phys. Chem. C.

[cit98] Zong X., Han J., Ma G., Yan H., Wu G., Li C. (2011). J. Phys. Chem. C.

[cit99] Chu J., Sun G., Han X., Chen X., Wang J., Hu W., Waluyo I., Hunt A., Du Y., Song B., Xu P. (2019). Nanoscale.

[cit100] Maeda K., Teramura K., Lu D., Takata T., Saito N., Inoue Y., Domen K. (2006). J. Phys. Chem. B.

[cit101] Tsuji E., Nanbu R., Degami Y., Hirao K., Watanabe T., Matsumoto N., Suganuma S., Katada N. (2020). Part. Part. Syst. Charact..

[cit102] Negishi Y., Matsuura Y., Tomizawa R., Kurashige W., Niihori Y., Takayama T., Iwase A., Kudo A. (2015). J. Phys. Chem. C.

[cit103] Wei L., Liu Z., Guo Z., Ruan M., Meng Y., Yan W. (2021). ACS Appl. Energy Mater..

[cit104] Qin Z., Chen Y., Wang X., Guo X., Guo L. (2016). ACS Appl. Mater. Interfaces.

[cit105] Meng S., Cui Y., Wang H., Zheng X., Fu X., Chen S. (2018). Dalton Trans..

[cit106] Guan S., Fu X., Zhang Y., Peng Z. (2018). Chem. Sci..

[cit107] Yin X. L., Li L. L., Jiang W. J., Zhang Y., Zhang X., Wan L. J., Hu J. S. (2016). ACS Appl. Mater. Interfaces.

[cit108] Liu X., Zhao Y., Yang X., Liu Q., Yu X., Li Y., Tang H., Zhang T. (2020). Appl. Catal. B Environ..

[cit109] Dong J., Shi Y., Huang C., Wu Q., Zeng T., Yao W. (2019). Appl. Catal. B Environ..

[cit110] Hong X., Yu X., Wang L., Liu Q., Sun J., Tang H. (2021). Inorg. Chem..

[cit111] Zhao Y., Lu Y., Chen L., Wei X., Zhu J., Zheng Y. (2020). ACS Appl. Mater. Interfaces.

[cit112] Wenderich K., Mul G. (2016). Chem. Rev..

[cit113] Wang Z., Inoue Y., Hisatomi T., Ishikawa R., Wang Q., Takata T., Chen S., Shibata N., Ikuhara Y., Domen K. (2018). Nat. Catal..

[cit114] Ma G., Liu J., Hisatomi T., Minegishi T., Moriya Y., Iwase M., Nishiyama H., Katayama M., Yamada T., Domen K. (2015). Chem. Commun..

[cit115] Li R., Zhang F., Wang D., Yang J., Li M., Zhu J., Zhou X., Han H., Li C. (2013). Nat. Commun..

[cit116] Vamvasakis I., Papadas I. T., Tzanoudakis T., Drivas C., Choulis S. A., Kennou S., Armatas G. S. (2018). ACS Catal..

[cit117] Liu W., Wang X., Yu H., Yu J. (2018). ACS Sustain. Chem. Eng..

[cit118] Dong Y., Kong L., Jiang P., Wang G., Zhao N., Zhang H., Tang B. (2017). ACS Sustain. Chem. Eng..

[cit119] Lu X., Ying Toe C., Ji F., Chen W., Wen X., Wong R. J., Seidel J., Scott J., Hart J. N., Ng Y. H. (2020). ACS Appl. Mater. Interfaces.

[cit120] Min S., Lei Y., Sun H., Hou J., Wang F., Cui E., She S., Jin Z., Xu J., Ma X. (2017). Mol. Catal..

[cit121] Di T., Zhu B., Zhang J., Cheng B., Yu J. (2016). Appl. Surf. Sci..

[cit122] Lu K.-Q., Qi M.-Y., Tang Z.-R., Xu Y.-J. (2019). Langmuir.

[cit123] Osterloh F. E. (2013). Chem. Soc. Rev..

[cit124] Hu R., Meng L., Zhang J., Wang X., Wu S., Wu Z., Zhou R., Li L., Li D. S., Wu T. (2020). Nanoscale.

[cit125] Pihosh Y., Minegishi T., Nandal V., Higashi T., Katayama M., Yamada T., Sasaki Y., Seki K., Suzuki Y., Nakabayashi M., Sugiyama M., Domen K. (2020). Energy Environ. Sci..

[cit126] Zhong X., He H., Du J., Ren Q., Huang J., Tang Y., Wang J., Yang L., Dong F., Bian L., Zhou Y. (2019). Electrochim. Acta.

[cit127] Bhat S. S. M., A Lee S., Lee T. H., Kim C., Park J., Lee T.-W., Kim S. Y., Jang H. W. (2020). ACS Appl. Energy Mater..

[cit128] He D., Gao R. T., Liu S., Sun M., Liu X., Hu K., Su Y., Wang L. (2020). ACS Catal..

[cit129] Gao R. T., Wu L., Liu S., Hu K., Liu X., Zhang J., Wang L. (2021). J. Mater. Chem. A.

[cit130] Wang L., Dionigi F., Truong Nguyen N., Kirchgeorg R., Gliech M., Grigorescu S., Strasser P., Schmuki P. (2015). Chem. Mater..

[cit131] Kanan M. W., Nocera D. G. (2008). Science.

[cit132] Zhang J., Huang Y., Lu X., Yang J., Tong Y. (2021). ACS Sustain. Chem. Eng..

[cit133] Haleem A. A., Majumder S., Perumandla N., Zahran Z. N., Naruta Y. (2017). J. Phys. Chem. C.

[cit134] Seo J., Takata T., Nakabayashi M., Hisatomi T., Shibata N., Minegishi T., Domen K. (2015). J. Am. Chem. Soc..

[cit135] Gao Y., Yang G., Dai Y., Li X., Gao J., Li N., Qiu P., Ge L. (2020). ACS Appl. Mater. Interfaces.

[cit136] Kim T. W., Choi K. S. (2014). Science.

[cit137] Wang L., Dionigi F., Truong Nguyen N., Kirchgeorg R., Gliech M., Grigorescu S., Strasser P., Schmuki P. (2015). Chem. Mater..

[cit138] Haleem A. A., Perumandla N., Naruta Y. (2019). ACS Omega.

[cit139] Chang J., Waclawik E. R. (2014). RSC Adv..

[cit140] Bakshi M. S. (2015). Cryst. Growth Des..

[cit141] Sakamoto N., Ohtsuka H., Ikeda T., Maeda K., Lu D., Kanehara M., Teramura K., Teranishi T., Domen K. (2009). Nanoscale.

[cit142] Maeda K., Sakamoto N., Ikeda T., Ohtsuka H., Xiong A., Lu D., Kanehara M., Teranishi T., Domen K. (2010). Chem. –Eur. J..

[cit143] Maeda K., Xiong A., Yoshinaga T., Ikeda T., Sakamoto N., Hisatomi T., Takashima M., Lu D., Kanehara M., Setoyama T., Teranishi T., Domen K. (2010). Angew. Chem., Int. Ed..

[cit144] Ikeda T., Xiong A., Yoshinaga T., Maeda K., Domen K., Teranishi T. (2012). J. Phys. Chem. C.

[cit145] Yoshinaga T., Saruyama M., Xiong A., Ham Y., Kuang Y., Niishiro R., Akiyama S., Sakamoto M., Hisatomi T., Domen K., Teranishi T. (2018). Nanoscale.

[cit146] Kawawaki T., Kataoka Y., Hirata M., Iwamatsu Y., Hossain S., Negishi Y. (2021). Nanoscale Horizons.

[cit147] Kim S., Nishino T., Saruyama M., Sakamoto M., Kobayashi H., Akiyama S., Yamada T., Domen K., Teranishi T. (2017). ChemNanoMat.

[cit148] Saruyama M., Kim S., Nishino T., Sakamoto M., Haruta M., Kurata H., Akiyama S., Yamada T., Domen K., Teranishi T. (2018). Chem. Sci..

[cit149] Su T., Shao Q., Qin Z., Guo Z., Wu Z. (2018). ACS Catal..

[cit150] Zhu J., Pang S., Dittrich T., Gao Y., Nie W., Cui J., Chen R., An H., Fan F., Li C. (2017). Nano Lett..

[cit151] Chen R., Fan F., Dittrich T., Li C. (2018). Chem. Soc. Rev..

[cit152] Tung R. T. (2014). Appl. Phys. Rev..

[cit153] Wang Z., Xue N., Chen J. (2019). J. Phys. Chem. C.

[cit154] Yoshida M., Yamakata A., Takanabe K., Kubota J., Osawa M., Domen K. (2009). J. Am. Chem. Soc..

[cit155] Yamakata A., Vequizo J. J. M. (2019). J. Photochem. Photobiol., C.

[cit156] Okano M., Sakamoto M., Teranishi T., Kanemitsu Y. (2014). J. Phys. Chem. Lett..

[cit157] Yamakata A., Kawaguchi M., Nishimura N., Minegishi T., Kubota J., Domen K. (2014). J. Phys. Chem. C.

[cit158] Toma F. M., Cooper J. K., Kunzelmann V., McDowell M. T., Yu J., Larson D. M., Borys N. J., Abelyan C., Beeman J. W., Yu K. M., Yang J., Chen L., Shaner M. R., Spurgeon J., Houle F. A., Persson K. A., Sharp I. D. (2016). Nat. Commun..

[cit159] Takashima T., Hashimoto K., Nakamura R. (2012). J. Am. Chem. Soc..

[cit160] Luo Z., Irtem E., Ibáñez M., Nafria R., Martí-Sánchez S., Genç A., de la Mata M., Liu Y., Cadavid D., Llorca J., Arbiol J., Andreu T., Ramon Morante J., Cabot A. (2016). ACS Appl. Mater. Interfaces.

[cit161] Zhang S., Zhang X., Rui Y., Wang R., Li X. (2021). Green Energy Environ..

[cit162] Zhang L., Jang H., Liu H., Kim M. G., Yang D., Liu S., Liu X., Cho J. (2021). Angew. Chem., Int. Ed..

[cit163] Fu L., Hu X., Li Y., Cheng G., Luo W. (2019). Nanoscale.

[cit164] Yoshida M., Takanabe K., Maeda K., Ishikawa A., Kubota J., Sakata Y., Ikezawa Y., Domen K. (2009). J. Phys. Chem. C.

[cit165] Takata T., Pan C., Nakabayashi M., Shibata N., Domen K. (2015). J. Am. Chem. Soc..

[cit166] Bau J. A., Takanabe K. (2017). ACS Catal..

[cit167] Okunaka S., Kameshige H., Ikeda T., Tokudome H., Hisatomi T., Yamada T., Domen K. (2020). ChemSusChem.

[cit168] Qureshi M., Shinagawa T., Tsiapis N., Takanabe K. (2017). ACS Sustain. Chem. Eng..

[cit169] Nishino T., Saruyama M., Li Z., Nagatsuma Y., Nakabayashi M., Shibata N., Yamada T., Takahata R., Yamazoe S., Hisatomi T., Domen K., Teranishi T. (2020). Chem. Sci..

[cit170] Khare R., Jentys A., Lercher J. A. (2020). Phys. Chem. Chem. Phys..

[cit171] Spanu D., Minguzzi A., Recchia S., Shahvardanfard F., Tomanec O., Zboril R., Schmuki P., Ghigna P., Altomare M. (2020). ACS Catal..

[cit172] Lee H., Yoon D. E., Koh S., Kang M. S., Lim J., Lee D. C. (2020). Chem. Sci..

[cit173] Zhang D. F., Zhang H., Guo L., Zheng K., Han X. D., Zhang Z. (2009). J. Mater. Chem..

[cit174] Xiong Z., Lei Z., Chen X., Gong B., Zhao Y., Zhang J., Zheng C., Wu J. C. S. (2017). Catal. Commun..

[cit175] Matsukawa M., Ishikawa R., Hisatomi T., Moriya Y., Shibata N., Kubota J., Ikuhara Y., Domen K. (2014). Nano Lett..

[cit176] Kuang Y., Jia Q., Ma G., Hisatomi T., Minegishi T., Nishiyama H., Nakabayashi M., Shibata N., Yamada T., Kudo A., Domen K. (2016). Nat. Energy.

